# Association of Percutaneous Endoscopic Gastrostomy (PEG) Tube Placement with Unplanned Hospitalization for Head and Neck Cancer

**DOI:** 10.3390/cancers17132066

**Published:** 2025-06-20

**Authors:** Sung Jun Ma, Jas Virk, Tyler V. Schrand, Jasmin Gill, Neil Almeida, Harshini K. Cheruvu, Vishal Gupta, Kimberly E. Wooten, Moni A. Kuriakose, Michael R. Markiewicz, Ryan P. McSpadden, Wesley L. Hicks, Mark K. Farrugia, Anurag K. Singh

**Affiliations:** 1 Department of Radiation Oncology, The Arthur G. James Cancer Hospital and Richard J. Solove Research Institute, The Ohio State University Comprehensive Cancer Center, 460 W 10th Ave, Columbus, OH 43210, USA; sungjun.ma@osumc.edu; 2 Department of Radiation Medicine, Roswell Park Comprehensive Cancer Center, 665 Elm Street, Buffalo, NY 14203, USA; tylerschrandz@gmail.com (T.V.S.); neil.almeida@roswellpark.org (N.A.); mark.farrugia@roswellpark.org (M.K.F.); 3 Jacobs School of Medicine and Biomedical Sciences, University at Buffalo, The State University of New York, 955 Main Street, Buffalo, NY 14203, USA; jasv@buffalo.edu; 4 Department of Biochemistry and Molecular Biology, Tulane University School of Medicine, 1430 Tulane Ave, New Orleans, LA 70112, USA; 5 University at Buffalo, The State University of New York, 12 Capen Hall, Buffalo, NY 14260, USA; jgill7@buffalo.edu; 6 Department of Statistics & Data Science, Cornell University, 1198 Comstock Hall, Ithaca, NY 14853, USA; hkc28@cornell.edu; 7 Department of Head & Neck/Plastic & Reconstructive Surgery, Roswell Park Comprehensive Cancer Center, 665 Elm Street, Buffalo, NY 14203, USA; vishal.gupta@roswellpark.org (V.G.); kimberly.wooten@roswellpark.org (K.E.W.); makuriakose@gmail.com (M.A.K.); mrm25@buffalo.edu (M.R.M.); ryan.mcspadden@roswellpark.org (R.P.M.); wesley.hicks@roswellpark.org (W.L.H.); 8 Department of Oral and Maxillofacial Surgery, School of Dental Medicine, University at Buffalo, The State University of New York, 3435 Main Street, Buffalo, NY 14214, USA

**Keywords:** head and neck cancer, percutaneous endoscopic gastrostomy, concurrent chemoradiation, dysphagia, odynophagia

## Abstract

Background/Objectives: There is a varying need for nutritional support among head and neck cancer (HNC) patients. Unplanned hospitalization is frequent with definitive chemoradiation. However, the association of unplanned hospitalizations with cancer control outcomes and percutaneous endoscopic gastrostomy (PEG) tube placement is not well-understood. This study aims to evaluate the clinical outcomes stratified by unplanned hospitalizations and to identify the prognostic factors associated with unplanned hospitalizations. Methods: This retrospective cohort study included 657 HNC patients treated with definitive chemoradiation at a single institution between 2007 and 2023. Relevant clinical data were evaluated for unplanned hospitalizations, prophylactic vs. therapeutic PEG tube placement, and clinical outcomes. Multivariable, subgroup, and matched-pair analyses were performed to account for potential confounding variables. The main outcomes and measures used are overall survival (OS), progression-free survival (PFS), locoregional failure (LRF), distant failure (DF), and incidence of unplanned hospitalization. Results: Unplanned hospitalizations occurred in 190 (29%) patients, which were associated with worse OS (adjusted hazards ratio [aHR] of 2.07, 95% confidence interval [CI] of 1.53–2.81, *p* < 0.001) and progression-free survival (aHR 1.83, 95% CI 1.38–2.41, *p* < 0.001). However, hospitalizations were not associated with LRF or DF outcomes. Similar findings were noted on 180 matched pairs as well as subgroups stratified by p16 status. In addition, when compared to patients with a prophylactic PEG tube, therapeutic PEG tube placement was associated with a higher risk of hospitalization (adjusted odds ratio [aOR] of 1.96, 95% CI 1.10–3.54, *p* = 0.02), while those without PEG tubes were less likely to be hospitalized (aOR 0.48, 95% CI 0.27–0.86, *p* = 0.01). Conclusions: Unplanned hospitalization was an independent, adverse prognostic factor for poor survival, but not oncologic outcomes. Unplanned hospitalization incidence was largely driven by those who required a therapeutic PEG tube, while it was the lowest for those who never needed a PEG tube.

## Simple Summary

This study aims to answer the following question: are there differences in the outcomes among head and neck cancer patients with prophylactic versus therapeutic percutaneous endoscopic gastrostomy (PEG) tubes? We found that unplanned hospitalizations were associated with poor survival, but not oncologic outcomes. Compared to patients with a prophylactic PEG tube, those without a PEG tube were less likely to be hospitalized, while others with a therapeutic PEG tube were more likely to be hospitalized. These results indicate that unplanned hospitalization is an independent, adverse prognostic factor for poor survival. The hospitalization incidence was the lowest among those who never needed PEG tubes, while those with therapeutic PEG tubes were most likely to be hospitalized.

## 1. Introduction

Even in the modern era of intensity-modulated radiation therapy (IMRT) routinely utilized for patients with head and neck cancer (HNC), nearly two thirds of these patients experience severe oral mucositis associated with unplanned hospitalization, weight loss, and frequent opioid use [1]. As a result, many studies suggest that having prophylactic percutaneous endoscopic gastrostomy (PEG) tubes may reduce risks of malnutrition and hospitalization, while minimizing treatment interruptions [2,3,4,5,6]. This contrasts with less than 10% of patients having a prophylactic PEG tube in a recent national cooperative group clinical trial [7]. The current cooperative-group phase III protocol (ClinicalTrials.gov ID: NCT05050162) and the Multinational Association of Supportive Care in Cancer (MASCC) guideline [8] did not specify algorithms for the use of prophylactic PEG tubes. The National Comprehensive Cancer Network (NCCN) guideline recommends an individualized approach for the use of prophylactic PEG tubes based on each patient’s functional and nutritional status at the treating physician’s discretion [9].

In addition to the differences in practice patterns regarding the use of prophylactic PEG tubes, many studies did not compare those with a therapeutic PEG tube versus others who did not need a PEG tube at all [2,3,4,5,6]. As a result, it remains unclear whether increased hospitalization rates were largely driven by a subset of patients without a PEG tube at baseline and how these rates were different from patients with a prophylactic PEG tube. Furthermore, the association between unplanned hospitalization and oncologic outcomes was not previously reported. To address this knowledge gap, we performed an observational cohort study to evaluate the association of unplanned hospitalization with clinical outcomes and PEG tube use.

## 2. Materials and Methods

Our study was reviewed and approved by the Institutional Review Board at the Roswell Park Comprehensive Cancer Center (EDR 103707), following the Strengthening the Reporting of Observational Studies in Epidemiology (STROBE) reporting guidelines.

Our single-institution database was queried for those diagnosed with non-metastatic HNC treated with definitive chemoradiation between June 2007 and April 2023. Everyone in our cohort underwent intensity-modulated radiation therapy with a total range of 69.96–70 Gy in 33–35 fractions [10]. Patients with metastatic cancer or treated with surgery, radiation alone, or palliative treatments were excluded.

Variables of interest included age, gender, race, smoking, primary cancer site, body mass index (BMI), cancer staging based on the American Joint Committee on Cancer (AJCC) 7th edition, p16 status, performance status, concurrent chemotherapy regimens, and PEG tube placement. Prophylactic PEG tube placement was defined as PEG tube placement prior to starting radiation, regardless of the patients’ baseline swallowing functions and nutritional status. Therapeutic PEG tube placement was defined as the PEG tube placement after starting radiation in the setting of a multidisciplinary evaluation based on patients’ swallowing function, nutritional status, significant weight loss, and severe mucositis pain among other factors. All variables were included for multivariable analysis (MVA) models, since they were considered clinically relevant. Missing values were coded as unknown prior to analysis.

Our primary endpoints were survival outcomes: overall survival (OS) and progression-free survival (PFS), defined as time intervals from diagnosis to death from any cause and to death from any cause or tumor progression, respectively. Other endpoints were locoregional failure (LRF) and distant failure (DF), defined as time intervals from diagnosis to tumor recurrence within and outside the head and neck, respectively. Another endpoint of interest was time to first unplanned hospitalization, defined as the time interval from the start of radiation to the date of unplanned admission.

### Statistical Analysis

Baseline variables were evaluated and compared based on the Fisher exact test and the Mann–Whitney U test for categorical and continuous characteristics, respectively. Reasons for unplanned hospitalization were summarized using descriptive statistics. OS and PFS were analyzed using the Kaplan–Meier plots, log-rank tests, and Cox proportional hazards MVA. LRF and DF were analyzed using the Fine–Gray MVA with death as a competing event and cumulative incidence plots. To reduce selection bias and evaluate the robustness of our MVA findings, propensity score matching was also performed comparing those with or without unplanned hospitalization. All variables were included for matching using the nearest neighbor method, 1:1 ratio without replacement, and a caliper distance of 0.2 [11]. Exploratory subgroup analysis was performed to analyze OS, PFS, LRF, and DF by repeating Cox and Fine–Gray MVA stratified by p16 status. Logistic MVA was performed to evaluate variables associated with first and multiple unplanned hospitalizations. Time to first hospitalization was analyzed using Fine–Gray MVA and cumulative incidence plots comparing those with or without a PEG tube placement. Given the multiple comparisons (therapeutic PEG tube vs. prophylactic PEG tube; no PEG tube vs. prophylactic PEG tube), Holm–Bonferroni method was performed in this setting. In addition, logistic MVA was also performed to identify the variables associated with therapeutic PEG tube placement among those without a PEG tube at baseline.

All *p* values were two-sided and those equal to or less than 0.05 were considered statistically significant. All analyses were performed using R version 4.3.2 (R Group for Statistical Computing).

## 3. Results

A total of 657 patients were identified and met our criteria (Table 1). Most patients were Caucasian (*n* = 568, 86.5%), male patients (*n* = 535, 81.4%) with a median age of 61.2 years (interquartile range: 54.9–67.0), former smoking history (*n* = 374, 56.9%), and oropharyngeal cancer (*n* = 375, 57.1%), who underwent definitive chemoradiation with cisplatin (*n* = 550, 83.7%). Median follow up was 30.3 months (interquartile range: 10.0–60.8).

**Figure 1:**
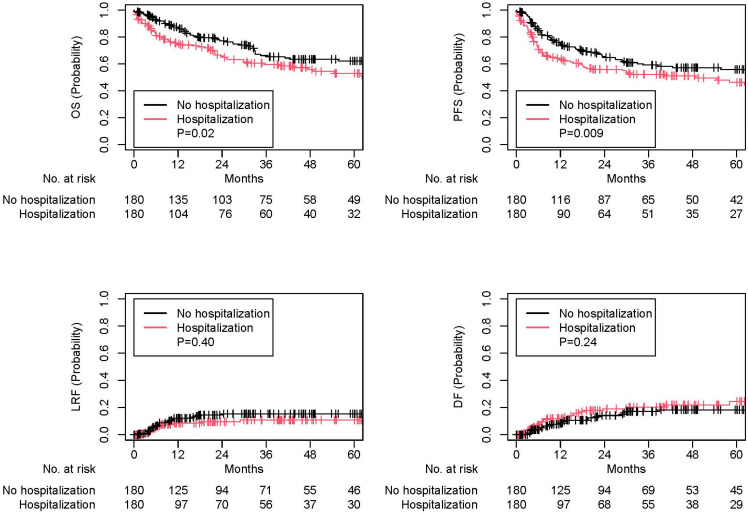
Kaplan–Meier and cumulative incidence plots for survival and tumor recurrence outcomes among the matched cohort. OS: overall survival; PFS: progression-free survival; LRF: locoregional failure; DF: distant failure.

Of 657 patients, 188 patients (28.6%) were hospitalized at least once and 52 patients (7.9%) were hospitalized more than once. Median duration of hospitalization was 5 days (interquartile range: 3–9). The three most common reasons for such unplanned hospitalizations were dehydration, fever, and dysphagia/odynophagia (Table 2). Those with unplanned hospitalization had a worse OS (adjusted hazards ratio [aHR] of 2.07, 95% confidence interval [CI] of 1.53–2.81, *p* < 0.001) and PFS (aHR 1.83, 95% CI 1.38–2.41, *p* < 0.001), but not LRF (aHR 0.78, 95% CI 0.43–1.42, *p* = 0.41) or DF (aHR 1.42, 95% CI 0.84–2.42, *p* = 0.19; Table S1). Similar findings were noted on 180 matched pairs (OS: HR 1.51, 95% CI 1.08–2.12, *p* = 0.02; PFS: HR 1.52, 95% CI 1.11–2.09, *p* = 0.009; LRF: HR 0.75, 95% CI 0.39–1.46, *p* = 0.40; DF: HR 1.38, 95% CI 0.80–2.37, *p* = 0.24; Figure 1 and Table 1) as well as subgroup cohorts for 263 patients with p16-negative tumors and 340 patients with p16-positive tumors (Table 3).

**Figure 2:**
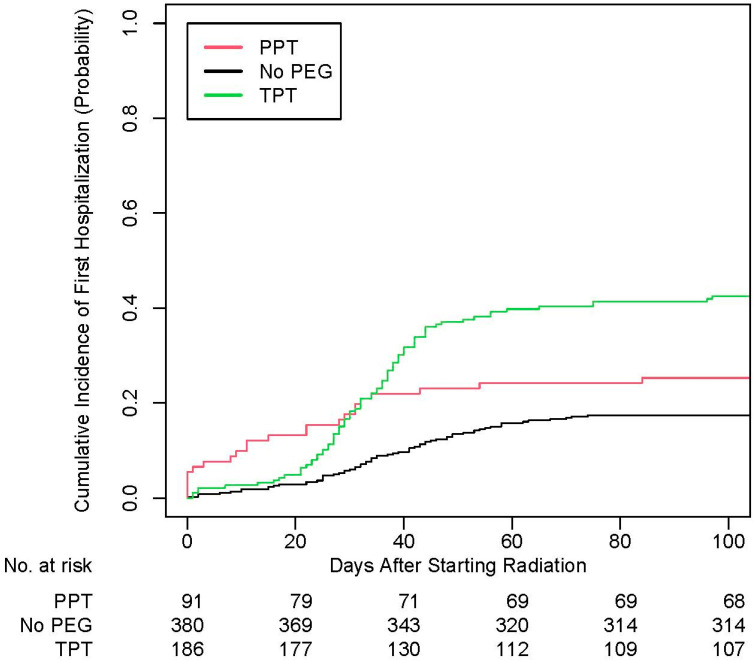
Cumulative incidence plot for time to first unplanned hospitalization. PPT: prophylactic percutaneous endoscopic gastrostomy tube; PEG: percutaneous endoscopic gastrostomy tube; TPT: therapeutic percutaneous endoscopic gastrostomy tube.

On logistic MVA for the entire cohort, male patients (adjusted odds ratio [aOR] of 0.46, 95% CI 0.29–0.72, *p* < 0.001) and those without a PEG tube (aOR 0.48, 95% CI 0.27–0.86, *p* = 0.01) were less likely to be hospitalized, while others with a therapeutic PEG tube (aOR 1.96, 95% CI 1.10–3.54, *p* = 0.02) were more likely to be hospitalized (Table 4). Similar findings were noted when evaluated for time to first unplanned hospitalization (Table 5 and Figure 2). In addition, those who were overweight were also less likely to be hospitalized multiple times (aOR 0.36, 95% CI 0.15–0.82, *p* = 0.02; Table 4). Among 566 patients without a prophylactic PEG tube, 186 patients (32.9%) received a therapeutic PEG tube. On logistic MVA among these patients, those with p16-positive tumors were less likely to receive therapeutic PEG tube placements (aOR 0.51, 95% CI 0.28–0.92, *p* = 0.03; Table 6).

## 4. Discussion

To our knowledge, this is the largest single-institution study suggesting that unplanned hospitalization is an independent, adverse prognostic factor for survival, but not cancer control outcomes, regardless of p16 status. Among those without a prophylactic PEG tube, more than two thirds of these patients did not require a therapeutic PEG tube and had the lowest incidence of unplanned hospitalization. Unplanned hospitalization was largely driven by those who required a therapeutic PEG tube.

The association between unplanned hospitalization and poor survival outcomes in our study was consistent with our prior report [12]. Such a finding was noted regardless of p16 status in our study, and it may be in part due to the poor overall clinical and nutritional status as indicated by the frequent need for therapeutic PEG tubes. However, unplanned hospitalization was not associated with poor oncologic outcomes in our study. In our comprehensive cancer center, daily radiation can be often continued on inpatients, and less than 1% of patients experienced radiation treatment interruptions in our prior study [13]. In addition, less than 10% of patients had multiple hospitalizations in our study, so a limited number of patients were at risk for missing multiple cycles of chemotherapy.

In our study, among those without a prophylactic PEG tube, approximately a third of patients required therapeutic PEG tube placement. This placement rate was consistent with other institutional studies reporting 20–40% of patients requiring therapeutic PEG tubes [3,14,15,16], though it was far less than over 60% reported in a recent national cooperative group trial [7]. This discrepancy may suggest significant heterogeneity in practice patterns for utilizing PEG tubes.

After stratifying patients without prophylactic PEG tubes into those who did or did not require therapeutic PEG tubes, unplanned hospitalization was mainly driven by those with a therapeutic PEG tube. However, other studies did not stratify those without a prophylactic PEG tube [2,4,5,6]. As a result, multiple studies reported no statistically significant differences in unplanned hospitalization due to the presence or absence of a prophylactic PEG tube [3,5,17]

A prophylactic PEG tube may be insufficient for preventing unplanned hospitalizations. For instance, other methods of nutritional supplementation could similarly address weight loss, [18] and frequent intravenous hydration may also help with pain control [19] There was also a high rate of non-use (47%) or limited use (less than two weeks) among those with a prophylactic PEG tube [20]. In addition, a prior review suggested that up to 40% of patients with a PEG tube may experience a varying severity of complications, such as PEG tube site infection, leakage of gastric contents, PEG tube dislodgement and blockage, and diarrhea from dietary changes [21,22].

Nonetheless, dehydration was the most common reason for hospitalization; therefore, unplanned hospitalizations could have been potentially reduced if those with therapeutic PEG tubes received prophylactic PEG tubes instead. Predicting which patients are at greatest risk of therapeutic PEG tube placement and unplanned hospitalizations may help. For example, a machine learning-based algorithm was previously shown to identify high-risk patients for hospitalization, [23] and an ongoing INSIGHT trial is currently investigating the role of close symptom surveillance for reducing hospitalizations among patients receiving definitive chemoradiation (ClinicalTrials.gov ID: NCT05338905). Volume de-escalation in radiation treatments has also been shown to numerically reduce the incidence of therapeutic PEG tubes within 3 months after treatment in a phase III clinical trial [24]. In addition, a secondary analysis of three prospective trials demonstrated that wearable devices allowed the continuous collection of daily step counts, and a machine learning-based model based on step counts predicted hospitalization during chemoradiation with accuracy, specificity, and sensitivity scores of 82.1%, 83.0%, and 60.7%, respectively [25]. A pilot trial incorporating wearable devices also showed that a week-to-week reduction in daily steps was associated with a substantial decline in quality of life [26].

In our study, p16-negative status was the only variable associated with therapeutic PEG tube placement among those without PEG tubes at baseline. Our finding is consistent with another study suggesting a greater disease burden and a higher likelihood of therapeutic PEG tube placement among those with p16-negative tumors [27]. However, our finding is inconsistent with another report showing that those with p16-positive tumors were associated with significant weight loss and therapeutic PEG tube placement as a result [16]. This discrepancy may be due to the higher nodal disease burden and combined modality treatments seen among those with p16-positive tumors [16].

Our study also showed that patients with overweight BMI were less likely to receive multiple hospitalizations. Such findings are consistent with another study reporting higher baseline BMI being associated with reduced complications, hospitalizations, and adequate nutritional status [28]. As a result, such patients may be able to have more cycles of chemotherapy, leading to improved survival outcomes [29,30]. Regardless of PEG tube use, overweight BMI has been similarly shown to be a favorable prognostic factor in patients with HNC undergoing definitive chemoradiation [13,31].

The limitations include the retrospective nature of our study. In addition, other clinically relevant variables, such as baseline functional and nutritional status, medical comorbidities, weight changes, adherence to treatments, toxicity profiles and their etiologies, dietary intake, the actual usage of a PEG tube by patients, exact duration of PEG tube placement, and complications from PEG tube placement, were unavailable for analysis. Our findings may not be generalizable to those who received surgery or had limited access to supportive care services during treatment. In addition, though a multidisciplinary discussion and shared decision-making occurred regarding nasogastric tube placement prior to finalizing therapeutic PEG tube placement, the nasogastric tube placement outcome was not collected for analysis in our database. Furthermore, radiation dosimetric data, such as radiation doses to pharyngeal constrictor muscles, were not available for all patients for analysis.

## 5. Conclusions

Our study showed that unplanned hospitalization is an independent, adverse prognostic factor for survival, but not cancer control outcomes, regardless of p16 status. Among those without a PEG tube at baseline, more than two thirds did not need a therapeutic PEG tube. Patients without PEG had the lowest incidence of unplanned hospitalization. Unplanned hospitalization was mainly driven by those who required a therapeutic PEG tube.

## Figures and Tables

**Table 1:** Baseline characteristics.

	Before Matching					After Matching				
	No Hospitalization		Hospitalization			No Hospitalization		Hospitalization		
	*n*	%	*n*	%	*p*	*n*	%	*n*	%	*p*
Age					0.03					0.66
<65 years	332	70.8	116	61.7		110	61.1	115	63.9	
65 years or older	137	29.2	72	38.3		70	38.9	65	36.1	
Gender					<0.001					0.71
Female	71	15.1	51	27.1		41	22.8	45	25.0	
Male	398	84.9	137	72.9		139	77.2	135	75.0	
Race					0.38					0.55
White	409	87.2	159	84.6		157	87.2	152	84.4	
Other	60	12.8	29	15.4		23	12.8	28	15.6	
Smoking					0.99					0.98
Never	121	25.8	48	25.5		47	26.1	48	26.7	
Former	266	56.7	108	57.4		100	55.6	101	56.1	
Current	82	17.5	32	17.0		33	18.3	31	17.2	
ECOG PS					0.85					0.39
0	125	26.7	52	27.7		41	22.8	49	27.2	
>0	344	73.3	136	72.3		139	77.2	131	72.8	
Primary site					0.65					0.92
Oropharynx	273	58.2	102	54.3		99	55.0	98	54.4	
Larynx	100	21.3	44	23.4		45	25.0	43	23.9	
Other	96	20.5	42	22.3		36	20.0	39	21.7	
BMI					0.25					1
Normal	114	24.3	41	21.8		41	22.8	41	22.8	
Underweight	13	2.8	8	4.3		8	4.4	8	4.4	
Overweight	181	38.6	62	33.0		62	34.4	61	33.9	
Obese	161	34.3	77	41.0		69	38.3	70	38.9	
T staging					0.49					0.4
1–2	247	52.7	93	49.5		97	53.9	88	48.9	
3–4	222	47.3	95	50.5		83	46.1	92	51.1	
N staging					0.63					0.91
0–1	126	26.9	54	28.7		51	28.3	53	29.4	
2–3	343	73.1	134	71.3		129	71.7	127	70.6	
HPV					0.6					0.72
Negative	182	38.8	81	43.1		79	43.9	77	42.8	
Positive	247	52.7	93	49.5		83	46.1	89	49.4	
Not available	40	8.5	14	7.4		18	10.0	14	7.8	
Cisplatin					0.91					0.67
No	76	16.2	31	16.5		27	15.0	31	17.2	
Yes	393	83.8	157	83.5		153	85.0	149	82.8	
PEG tube placement					<0.001					0.91
Prophylactic	63	13.4	28	14.9		30	16.7	28	15.6	
No	307	65.5	73	38.8		69	38.3	73	40.6	
Therapeutic	99	21.1	87	46.3		81	45.0	79	43.9	

PEG tube: percutaneous endoscopic gastrostomy tube; ECOG PS: Eastern Cooperative Oncology Group performance status; BMI: body mass index; HPV: human papillomavirus.

**Table 2:** Reasons for hospitalization.

	Reasons for Hospitalization	
	*n*	%
Dehydration	58	19.9
Fever	41	14.1
Dysphagia or odynophagia	27	9.3
Pneumonia	24	8.2
Altered mental status	10	3.4
PE or DVT	7	2.4
Other	124	42.6
Total	291	100.0

PE: pulmonary embolism; DVT: deep vein thrombosis.

**Table 3:** Cox and Fine–Gray multivariable analysis for survival and tumor recurrence outcomes stratified by p16 status.

p16-Negative Tumors												
	Overall survival			Progression-free survival			Locoregional failure			Distant failure		
	aHR	95% CI	*p*	aHR	95% CI	*p*	aHR	95% CI	*p*	aHR	95% CI	*p*
Hospitalization												
No	Reference			Reference			Reference			Reference		
Yes	1.92	1.24–2.97	0.003	1.84	1.24–2.74	0.002	0.83	0.40–1.73	0.62	1.05	0.46–2.39	0.91
p16-positive tumors												
	Overall survival			Progression-free survival			Locoregional failure			Distant failure		
	aHR	95% CI	*p*	aHR	95% CI	*p*	aHR	95% CI	*p*	aHR	95% CI	*p*
Hospitalization												
No	Reference			Reference			Reference			Reference		
Yes	2.47	1.38–4.40	0.002	1.8	1.11–2.92	0.02	0.25	0.02–2.77	0.26	1.44	0.65–3.16	0.37

aHR: adjusted hazards ratio; CI: confidence interval.

**Table 4:** Logistic multivariable analysis for first and multiple unplanned hospitalizations.

	First Hospitalization			Multiple Hospitalization		
	aOR	95% CI	*p*	aOR	95% CI	*p*
PEG tube placement						
Prophylactic	Reference			Reference		
No	0.48	0.27–0.86	0.01	0.86	0.35–2.31	0.76
Therapeutic	1.96	1.10–3.54	0.02	1.81	0.74–4.81	0.21
Age						
For every increase by 1	1.01	0.99–1.03	0.2	1.01	0.98–1.05	0.39
Gender						
Female	Reference			Reference		
Male	0.46	0.29–0.72	<0.001	0.6	0.31–1.23	0.15
Race						
White	Reference			Reference		
Other	1.32	0.77–2.21	0.3	0.53	0.17–1.30	0.2
Smoking						
Never	Reference			Reference		
Former	1.03	0.66–1.62	0.88	0.87	0.42–1.85	0.7
Current	0.91	0.49–1.65	0.75	0.71	0.26–1.88	0.49
ECOG PS						
0	Reference			Reference		
>0	0.8	0.53–1.21	0.29	2.24	1.02–5.62	0.06
Primary site						
Oropharynx	Reference			Reference		
Larynx	1.23	0.64–2.38	0.53	1.34	0.47–3.79	0.58
Other	1.24	0.70–2.18	0.45	1.37	0.54–3.31	0.49
BMI						
Normal	Reference			Reference		
Underweight	1.31	0.45–3.67	0.6	1.19	0.24–4.52	0.81
Overweight	0.98	0.59–1.65	0.95	0.36	0.15–0.82	0.02
Obese	1.54	0.92–2.60	0.1	0.71	0.33–1.54	0.39
T staging						
1–2	Reference			Reference		
3–4	1.01	0.67–1.50	0.97	1.13	0.59–2.17	0.72
N staging						
0–1	Reference			Reference		
2–3	1.02	0.63–1.65	0.94	0.77	0.37–1.63	0.49
HPV						
Negative	Reference			Reference		
Positive	1.24	0.70–2.20	0.45	1.47	0.59–3.68	0.41
Not available	0.89	0.38–2.04	0.79	1.52	0.39–5.37	0.53
Cisplatin						
No	Reference			Reference		
Yes	1.09	0.65–1.84	0.75	1.34	0.59–3.41	0.51

aOR: adjusted odds ratio; CI: confidence interval; PEG tube: percutaneous endoscopic gastrostomy tube; ECOG PS: Eastern Cooperative Oncology Group performance status; BMI: body mass index; HPV: human papillomavirus.

**Table 5:** Fine–Gray multivariable analysis for time to first unplanned hospitalization.

	Time to First Hospitalization		
	aHR	95% CI	*p*
PEG tube placement			
Prophylactic	Reference		
No	0.52	0.32–0.85	0.008
Therapeutic	1.59	1.00–2.54	0.05
Age			
For every increase by 1	1.01	0.99–1.03	0.18
Gender			
Female	Reference		
Male	0.57	0.41–0.80	0.001
Race			
White	Reference		
Other	1.17	0.78–1.76	0.45
Smoking			
Never	Reference		
Former	1.01	0.70–1.45	0.95
Current	0.91	0.55–1.52	0.73
ECOG PS			
0	Reference		
>0	0.84	0.61–1.16	0.29
Primary site			
Oropharynx	Reference		
Larynx	1.18	0.71–1.97	0.52
Other	1.14	0.73–1.77	0.58
BMI			
Normal	Reference		
Underweight	1.21	0.54–2.70	0.64
Overweight	0.94	0.62–1.41	0.75
Obese	1.27	0.84–1.92	0.26
T staging			
1–2	Reference		
3–4	1.01	0.73–1.38	0.98
N staging			
0–1	Reference		
2–3	1.01	0.70–1.47	0.95
HPV			
Negative	Reference		
Positive	1.22	0.78–1.91	0.38
Not available	0.97	0.47–2.00	0.93
Cisplatin			
No	Reference		
Yes	1.05	0.67–1.63	0.84

aHR: adjusted hazards ratio; CI: confidence interval; PEG tube: percutaneous endoscopic gastrostomy tube; ECOG PS: Eastern Cooperative Oncology Group performance status; BMI: body mass index; HPV: human papillomavirus.

**Table 6:** Logistic multivariable analysis for therapeutic percutaneous endoscopic gastrostomy (PEG) tube placement among those without a PEG tube at baseline.

	aOR	95% CI	*p*
Age			
For every increase by 1	1.01	0.99–1.03	0.25
Gender			
Female	Reference		
Male	0.93	0.58–1.54	0.79
Race			
White	Reference		
Other	0.69	0.37–1.25	0.24
Smoking			
Never	Reference		
Former	0.84	0.54–1.32	0.45
Current	1.22	0.66–2.23	0.52
ECOG PS			
0	Reference		
>0	1.35	0.90–2.06	0.15
Primary site			
Oropharynx	Reference		
Larynx	0.68	0.34–1.34	0.26
Other	0.71	0.39–1.26	0.25
BMI			
Normal	Reference		
Underweight	2.26	0.60–8.78	0.22
Overweight	1.47	0.88–2.49	0.15
Obese	1.25	0.74–2.15	0.41
T staging			
1–2	Reference		
3–4	1.36	0.91–2.02	0.13
N staging			
0–1	Reference		
2–3	1.15	0.70–1.90	0.58
HPV			
Negative	Reference		
Positive	0.51	0.28–0.92	0.03
Not available	1.6	0.67–3.88	0.29
Cisplatin			
No	Reference		
Yes	1.19	0.71–2.03	0.53

aOR: adjusted odds ratio; CI: confidence interval; PEG tube: percutaneous endoscopic gastrostomy tube; ECOG PS: Eastern Cooperative Oncology Group performance status; BMI: body mass index; HPV: human papillomavirus.

## Data Availability

The data underlying this article cannot be shared publicly for the privacy of individuals that participated in this study.

## Supplementary Materials

The following supporting information can be downloaded at: https://www.mdpi.com/article/10.3390/cancers17132066/s1, Table S1: Cox and Fine-Gray multivariable analysis for survival and tumor recurrence outcomes.
