# Supplementary material for: Association of Percutaneous Endoscopic Gastrostomy (PEG) Tube Placement with Unplanned Hospitalization for Head and Neck Cancer
Source: Cancers (Basel). 2025 Jun 20;17(13):2066. doi: 10.3390/cancers17132066 (PMC12249407; doi:10.3390/cancers17132066)
Supplement: Supplementary file 1 [file cancers-17-02066-s001.zip › cancers-3663457-supplementary.pdf]

Supplemental Table

Table S1. Cox and Fine-Gray multivariable analysis for survival and tumor recurrence outcomes

Table S1. Cox and Fine-Gray multivariable analysis for survival and tumor recurrence outcomes

	Overall survival			Progression-free survival			Locoregional failure			Distant failure		
	aHR	95% CI	P	aHR	95% CI	P	aHR	95% CI	P	aHR	95% CI	P
Hospitalization												
No	Reference			Reference			Reference			Reference		
Yes	2.07	1.53-2.81	<0.001	1.83	1.38-2.41	<0.001	0.78	0.43-1.42	0.41	1.42	0.84-2.42	0.19
PEG tube placement												
Prophylactic	Reference			Reference			Reference			Reference		
No	0.84	0.56-1.24	0.37	0.81	0.56-1.16	0.25	0.71	0.36-1.41	0.32	0.73	0.35-1.50	0.39
Therapeutic	0.79	0.53-1.17	0.24	0.87	0.60-1.24	0.43	0.57	0.27-1.20	0.14	1.1	0.55-2.21	0.78
Age												
For every increase by 1	1.02	1.00-1.04	0.02	1.01	1.00-1.03	0.16	1	0.97-1.03	0.93	1.01	0.98-1.03	0.66
Gender												
Female	Reference			Reference			Reference			Reference		
Male	1.41	0.98-2.03	0.07	1.42	1.02-1.98	0.04	1.07	0.58-1.99	0.83	2.96	1.33-6.57	0.008
Race												

White	Reference			Reference			Reference			Reference		
Other	1.19	0.81-1.75	0.36	1.11	0.78-1.58	0.57	1.23	0.63-2.38	0.55	0.84	0.42-1.68	0.63
Smoking												
Never	Reference			Reference			Reference			Reference		
Former	1.48	0.99-2.19	0.05	1.37	0.97-1.94	0.07	1.5	0.73-3.08	0.27	1.44	0.80-2.57	0.22
Current	2.00	1.26-3.17	0.003	1.58	1.04-2.39	0.03	1.81	0.81-4.01	0.15	1.18	0.55-2.51	0.67
ECOG PS												
0	Reference			Reference			Reference			Reference		
>0	1.37	0.97-1.93	0.08	1.38	1.01-1.89	0.05	1.15	0.61-2.16	0.67	1.41	0.84-2.37	0.19
Primary site												
Oropharynx	Reference			Reference			Reference			Reference		
Larynx	1.17	0.70-1.93	0.55	0.97	0.61-1.53	0.89	1.49	0.51-4.34	0.46	1.1	0.53-2.26	0.8
Other	1.54	0.97-2.44	0.06	1.36	0.91-2.03	0.13	2.99	1.27-7.04	0.01	1.7	0.90-3.20	0.1
BMI												
Normal	Reference			Reference			Reference			Reference		
Underweight	1.75	0.97-3.16	0.06	1.45	0.81-2.61	0.21	1.82	0.70-4.74	0.22	2.68	1.05-6.89	0.04

Overweight	0.65	0.46-0.93	0.02	0.6	0.43-0.84	0.003	0.48	0.25-0.94	0.03	0.87	0.47-1.60	0.65
Obese	0.66	0.45-0.98	0.04	0.69	0.49-0.97	0.03	0.75	0.39-1.45	0.39	0.79	0.41-1.50	0.47
T staging												
1-2	Reference			Reference			Reference			Reference		
3-4	2.20	1.60-3.02	<0.001	1.87	1.41-2.47	<0.001	2.43	1.33-4.43	0.004	2.87	1.75-4.68	<0.001
N staging												
0-1	Reference			Reference			Reference			Reference		
2-3	1.45	1.02-2.05	0.04	1.45	1.05-1.99	0.02	0.95	0.53-1.70	0.86	2.64	1.43-4.85	0.002
HPV												
Negative	Reference			Reference			Reference			Reference		
Positive	0.79	0.51-1.22	0.29	0.71	0.48-1.06	0.09	0.78	0.31-1.95	0.6	1.03	0.55-1.92	0.94
Not available	1.22	0.69-2.15	0.5	0.88	0.52-1.49	0.64	1.45	0.44-4.79	0.54	0.62	0.23-1.69	0.35
Cisplatin												
No	Reference			Reference			Reference			Reference		
Yes	0.65	0.44-0.95	0.02	0.65	0.47-0.92	0.01	1.12	0.51-2.48	0.78	0.62	0.35-1.11	0.11

aHR: adjusted hazards ratio; CI: confidence interval; PEG tube: percutaneous endoscopic gastrostomy tube; ECOG PS: Eastern Cooperative Oncology Group performance status; BMI: body mass index; HPV: human papillomavirus
